# Autoimmune Cytopenias in Chronic Lymphocytic Leukemia, Facts and Myths

**DOI:** 10.4084/MJHID.2013.068

**Published:** 2013-11-04

**Authors:** Pavankumar Tandra, Jairam Krishnamurthy, Vijaya Raj Bhatt, Kam Newman, James O Armitage, Mojtaba Akhtari

**Affiliations:** 1 Division of Hematology and Oncology, University of Nebraska Medical Center, Omaha, NE 68198, USA.; 2 Dr. Kam Newman, Section of Transfusion Medicine, Cleveland Clinic Foundation, 9500 Euclid Avenue 6-1, Cleveland, OH 44195, USA.

## Abstract

CLL has been defined as presence of more than 5000 small mature appearing monoclonal B lymphocytes with a specific immunophenotype in peripheral blood. It is a well-known fact that CLL is associated with autoimmune cytopenias. CLL cells are CD5^+^ B lymphocytes, and usually are not the “guilty” cells which produce autoantibodies. T cell defect is another characteristic of CLL and the total number of T cells is increased, and there is inversion of the CD4/CD8 ratio. Autoimmune hemolytic anemia (AIHA) is the most common autoimmune complication of CLL and has been reported in 10–25% of CLL patients. However, the stage-adjusted estimated rate of AIHA in CLL is about 5%. Conversely, CLL is three times more common in patients who present with AIHA. Direct agglutinin test (DAT) is positive in 7–14% of CLL patients but AIHA may also occur in DAT negative patients.

Autoimmune thrombocytopenia (AIT) is the second most common complication of CLL and has been reported in 2–3% of patients. DAT is positive in AIT but presence of antiplatelet antibodies is neither diagnostic nor reliable. Autoimmune neutropenia (AIN) and pure red cell aplasia (PRCA) are very rare complications of CLL and like other autoimmune complications of CLL may occur at any clinical stage. It is believed that most case reports of AIN and PRCA in CLL actually belong to large granular lymphocytic leukemia (LGL). Non-hematologic autoimmune complications of CLL including cold agglutinin disease (CAD), paraneoplastic pemphigus (PNP), acquired angioedema, and anti-myelin associated globulin are rare.

Before starting any treatment, clinicians should distinguish between autoimmune cytopenias and massive bone marrow infiltration since autoimmune complications of CLL are not necessarily equal to advanced disease with poor prognosis. According to IWCLL guideline, steroids are the mainstay of treatment of simple autoimmunity. Intravenous immunoglobulin (IVIg), cyclosporine, and rituximab are used in complex, steroid refractory cases. Monotherapy with purine analogues and alkylating agents should be avoided as they may increase CLL associated autoimmune complications.

## Introduction

Chronic lymphocytic leukemia (CLL), characterized by progressive accumulation of nonfunctional and monoclonal B lymphocytes in the blood, bone marrow and lymphatic system,^1^ is the most common leukemia in the western world. CLL accounts for approximately 30 percent of all leukemias.^2^ According to the National Cancer Institute-Working Group (NCI-WG) 2008, CLL is presence of greater than 5000 small mature appearing monoclonal B lymphocytes in the peripheral blood. However, the clonality of B lymphocytes has to be confirmed by flow cytometry. CLL is mainly a disease of elderly and the median age at onset is 72 years. As it is evident from data of 18 Surveillance Epidemiology and End Results (SEER) databases, the age-adjusted incidence rate for CLL between the years of 2005–2009 was 4.2 per 100,000 men and women annually.^3^

Autoimmunity secondary to CLL may have hematologic and non-hematologic manifestations.^4^,^5^ Hematologic autoimmune phenomena include hemolytic anemia (AIHA), thrombocytopenia (AIT), and neutropenia (AIN), and pure red blood cell aplasia (PRCA).

Autoimmune cytopenias in CLL may occur at any stages of CLL, respond well to treatment and do not affect the overall survival of CLL patients.^6^ Although a number of non-hematologic autoimmune conditions have sporadic associations with CLL, autoimmune paraneoplastic pemphigus, autoimmune glomerulonephritis and autoimmune C1 esterase inhibitor deficiency have been shown to have a definite association^1^,^6^ (Tables 1 and 2 ).

## Epidemiology of Autoimmune Cytopenias

The incidence of autoimmune cytopenias varies from 4.3% to 26 % in different reports.^6^ Since these data has been extracted from tertiary care centers database, the true prevalence and incidence of autoimmune cytopenias in CLL patients is unknown. Prior studies might have overestimated the prevalence owing to the lack of specific diagnostic methods. On the other hand, better management and new drugs that have changed the overall survival of these patients, have affected the prevalence of autoimmune cytopenias in CLL patients. Autoimmune neutropenia might have been over reported in some of previous series since these studies have included large granular lymphocytic leukemia (LGL) in their study.^7^ Recent studies estimated that the incidence of autoimmune cytopenias might be in the range of 5 to 10%.^8^

A study of 1750 CLL patients seen over a period of 10 years at Mayo clinic^9^ found that 24% of patients had cytopenias. The most common causes of cytopenias were marrow failure (54%), autoimmunity (18%), non CLL related cytopenias (11%), long term complications of treatment (4%) and splenomegaly (3%).

The incidence of AIT was estimated 1–5% in various studies.^9^–^11^ The AIN incidence is much more difficult to assess since neutropenia is frequently not CLL-related, and currently available tests (anti neutrophil antibodies) cannot diagnose AIN with certainty. The incidence of AIN in a large series of CLL patients followed for 10 years at Mayo clinic, was 0.17 % (3 out of 1750).^12^

A thorough review of literature using words “auto immune granulocytopenia”, “auto immune leukopenia” “autoimmune neutropenia” in CLL revealed only 4 case reports of CLL associated neutropenia.^13^,^14^ All of these case reports were autoimmunity secondary to monotherapy with purine analogues or alkylating agents.

## Pathogenesis of Autoimmune Cytopenias in CLL

### A brief overview of autoimmunity

Autoimmune diseases are caused by adaptive immunity where, immune responses are directed towards autologous component(s) of the body. They represent loss of the self-tolerance mechanisms in circulating B and T cells. A defining characteristic of autoimmune diseases is the presence of antibodies and T cells specific for antigens expressed by the target tissue. These antigens are called auto antigens; the effectors of adaptive immunity that recognize them are known as autoantibodies and autoimmune T cells. There are regulatory mechanisms in the T cell repertoire where auto reactive T cells are usually suppressed by regulatory T cells (Treg) and imbalance of these mechanisms can result in autoimmunity.^15^–^19^

Autoimmune diseases can be classified by the type of adaptive immunological effector mechanism causing the disease. Three kinds of mechanisms are responsible which correspond to three of the four categories of hypersensitivity reactions (types II, III IV). Autoimmune diseases are never caused by immunoglobulin E (IgE), the source of type I hypersensitivity reactions.^15^ Description of these effector mechanisms is beyond the scope of this review.

### Mechanisms of autoimmunity in CLL

In autoimmune cytopenias, autoimmunity usually corresponds to the type II hypersensitivity reaction in which the antibodies are frequently directed at the antigens on blood cells. Immunoglobulin G and IgM antibodies bind to certain blood cells (red cells, platelets and neutrophils), where they activate complement system by classical pathway, and trigger their destruction. Alternatively, phagocytes in reticuloendothelial system may clear C3b and antibody-coated cells from circulation.^15^

The underlying mechanisms of pathologic auto antibody production in CLL associated cytopenias are not well understood. In AIHA, both the T cell dysfunction and malignant B cells are involved in autoantibody production. Malignant B lymphocytes act as antigen presenting cells (APCs) and present antigen to T Helper cell and lead to their activation. Activated T helper cells mediate antibody production by non-malignant B lymphocytes. These auto antibodies lead to hemolysis by destroying the target red cells. Murine and human studies revealed these antibodies are mostly targeted towards Rh antigens on the surface of red blood cells.^20^,^21^

### Autoimmune thrombocytopenia

There are many proposed mechanisms that by which autoantibodies are produced and interact with antigens on platelets in CLL. Ninety percent of auto antibodies are produced by nonmalignant B cells rather than malignant B cells^22^ and are directed towards platelet surface antigens glycoprotein Ib/IX and IIb/IIIa.^18^,^19^ These autoantibodies are IgG type and polyclonal in nature. These circulating autoantibodies attach to the platelet antigens and destroy the platelets via opsonisation and antibody dependent cellular cytotoxicity and are finally cleared in the reticuloendothelial system in the liver and spleen. In 10 % of the cases, the mechanism is similar to cold hemagglutinin disease where the IgM monoclonal antibodies are produced by malignant B cells.^23^ Besides production of pathologic autoantibodies, there is evidence to suggest that suppression of Treg cells also leads to autoimmunity in CLL which was first reported in post-fludarabine cytopenias.^16^–^19^

Visco et al retrospectively studied 463 CLL patients with available immunoglobulin heavy-chain variable (IGHV) gene status and B-cell receptor (BCR) configuration [heavy-chain complementary-determining region 3 (HCDR3)], of whom 36 developed AIT. Unmutated IGHV mutational status, deletion 11q23 and stereotyped BCR were significantly associated with shorter time to AIT development than other factors (). 24,25 The risk of AIT has been reported to be increased in CLL patients with positive cells for ZAP70 expression, negative for CD38 and abnormal fluorescence in situ hybridization (FISH).^11^,^26^–^28^ Positive direct anti globulin test and occurrence of AIHA are other reported risk factors for AIT development.^11^

Anti-platelet antibodies may present in CLL patients without having thrombocytopenia. The importance of this phenomenon is not clear and there is no data on how many of these patients subsequently will develop thrombocytopenia. With combination of autoantibody mediated megakaryocyte destruction and inability of the marrow to produce megakaryocytes secondary to infiltrating leukemic cells AIT develops.^8^

### Autoimmune neutropenia

Pathogenesis of AIN is not well studied and our current understanding of autoimmune neutrophil destruction is mainly based on *in vitro* observations. Agglutination of neutrophils due to anti neutrophil antibodies (ANAs), complement mediated neutrophil destruction and phagocytosis of neutrophils coated with ANAs in spleen and liver is some of the proposed mechanisms.^29^

Autoimmune neutropenia is commonly seen in association with connective tissue disorders, Grave’s disease, *hepatitis B and C*, human immunodeficiency virus, parvovirus B19, and *Helicobacter pylori* infections, and malignancies such as large granular lymphocytic leukemia, hairy cell leukemia and Hodgkin’s lymphoma. Some of these conditions may coexist with CLL, and should be considered before labeling CLL as the culprit for AIN.^29^ Autoimmune neutropenia has been commonly seen in hairy cell leukemia and Hodgkin’s Lymphoma but is rare in CLL.^23^,^30^ Mono therapy of CLL with medications such as fludarabine, rituximab and alemtuzumab are also associated with AIN.^14^,^31^

## Clinical Presentation

### Autoimmune thrombocytopenia

Autoimmune thrombocytopenia is an incidental finding in more than half of CLL patients^12^,^32^ and it is prudent that patients with immune thrombocytopenia be screened for CLL.^33^,^34^ In an Italian series, the median time from diagnosis of CLL to development of AIT was 13 months.^11^ Thrombocytopenia due to marrow infiltration is usually seen in later stages but AIT may occur at any time during the course of CLL.^33^,^34^

Bleeding due to thrombocytopenia is rare in CLL associated AIT unless platelet counts are very low (less than 15,000). Even in rapid AIT, only 50% of patients present with bleeding and less than 10% has clinically significant bleeding.^12^

### Autoimmune Neutropenia

Recurrent infections are the only clinical presentation of AIN and should be identified early and treated appropriately. The classic signs and symptoms of infection may be absent in neutropenia. The risk of infection is high with an absolute neutrophil count (ANC) below 500 and it increases exponentially with an ANC below 100 for more than 5 days.^35^–^37^

Common sites of infection in severe neutropenia (ANC below 500) include skin, oral cavity, and perirectal area in addition to intravenous lines and port-a-catheters. Careful examination of all catheter sites is part of AIN evaluation. In Elting *et al* study, gram positive organisms comprise 46% of the infections, while 42% are due to gram negative organisms and 12% are poly microbial; lung (40%), and skin and soft tissues (30%) were the most common sites and urinary tract, sinuses and oropharynx, skeletal, enteric tract, meninges, and endocardium were the remaining cases.^35^

## Diagnosis

Gradual cytopenias in CLL are multifactorial. If CLL associated cytopenia occurs acutely, sepsis syndrome should be considered since CLL patients are at increased risk of opportunistic infections owing to nonfunctional lymphocytes and related hypogammaglobulinemia.

A high degree of suspicion is required to diagnose autoimmunity as a cause of cytopenias given the multiple etiologies. Cytopenias might present before CLL requires therapy, however, the highest risk is in patients with advanced disease.^38^ Clinicians should suspect AIT when there is more than 50% drop in platelet count to lower than 100000. If platelet counts were low from the beginning, then a careful examination of the peripheral blood smear is required.^39^ An assay for EDTA dependent pseudo thrombocytopenia and inspection of platelet volume curve to rule out inherited macro thrombocytopenia may also be required. Furthermore, a careful review of recent changes in medications including prescribed and over the counter drugs is useful to exclude drug induced thrombocytopenia. Platelet antibodies are neither sensitive nor specific for AIT^18^,^40^ and their confirmation is not necessary for diagnosis of CLL associated AIT.^41^ Increased mean platelet volume and platelet distribution width are suggestive of AIT in some patients.

In advanced CLL, isolated thrombocytopenia without anemia is more likely to have autoimmune etiology.^33^,^34^,^42^ Because thrombocytopenia commonly seen with infections such as Hepatitis C, *Helicobacter pylori* and human immunodeficiency virus, it is prudent to investigate for these infections, and sometimes management of these infections may increase platelet counts without using corticosteroids^43^ (Table 3).

Demonstration of increased and reticulated megakaryocytes in bone marrow in response to thrombocytopenia confirms the diagnosis. Bone marrow biopsy shows normal or increased megakaryocytes with immature forms. Sometimes heavily infiltrated bone marrow with leukemic cells makes it difficult to assess the megakaryocyte numbers. Response to the trial of intravenous immunoglobulin or steroids may confirm the diagnosis. Thirty percent of AIT cases also have simultaneous AIHA (Evans Syndrome).^44^,^45^

In contrast, AIN is extremely rare and difficult to diagnose with certainty.^29^ AIN is a diagnosis of exclusion and only should be suspected when there is persistent and prolonged absolute neutropenia accompanied with failure of neutrophil production or maturation arrest in bone marrow.^5^ Antineutrophil antibodies are neither sensitive nor specific and should not be used as a tool for diagnosis. It should be emphasized that it is difficult to appreciate neutrophil precursors in a heavily infiltrated marrow by leukemic cells. It is crucial to recognize marrow infiltration as soon as possible since these patients are at risk for overwhelming infections and sepsis^5^ (Table 4).

## Management

### Autoimmune thrombocytopenia

There is no randomized controlled clinical trial for management of CLL associated autoimmune thrombocytopenia, and treatment is mainly based on small case series. Whether patient requires CLL chemotherapy or autoimmune disorder management is the first question that clinicians should raise.

In patients with non-progressive CLL, management is similar to primary immune thrombocytopenic purpura with corticosteroids, Intravenous immunoglobulin, rituximab, thrombopoietin receptor agonists, and splenectomy in refractory cases. In a life threatening hemorrhage condition that requires a rapid intervention, intravenous immunoglobulin 1 gram per kilogram per day^43^ or methyl prednisone 1 gram per day for 3 doses, followed by platelet transfusion may be lifesaving.^4^ Intravenous immunoglobulin effect does not last for more than a few weeks in the body.

According to the American society of hematology guideline, if thrombocytopenia is an incidental finding and there is no significant hemorrhage, there is no need for treatment. However, treatment is indicated when platelet count drops below 30,000, and is low enough to constitute a risk of bleeding.^43^

### Corticosteroids

Oral prednisone, dexamethasone and intravenous methyl prednisolone have shown equal efficacy in the treatment of AIT. Oral prednisone at a dose of 0.5 to 2 mg per kilogram body weight should be initiated and tapered after a response has been achieved. It usually takes 1–2 weeks to observe a response. However, if there is no response after 4–6 weeks, it is highly unlikely to respond to steroids and alternative treatments should be sought. Pulse therapy with high dose dexamethasone (40 milligrams per day for 4 days which can be repeated every couple of weeks) is another option.^47^

More than two third of patients will completely respond to steroids, but there is no response in the remained one third of patients. In such a condition, any of the other therapies described below can be tried. Among responders, the median duration of response may have about 2 years.^12^

### Immunosuppressive agents other than corticosteroids

Low dose cyclosporine,^48^ mycophenolate mofetil,^49^,^50^ cyclophosphamide^51^,^52^ or azathioprine are alternative immunosuppressive therapy^53^ and may be considered in patients who fail to respond to corticosteroids, require higher maintenance doses, relapse quickly or who develop side effects (Table 5).

### Autoimmune thrombocytopenia refractory to immunosuppression

The goal of treatment for CLL associated AIT refractory to corticosteroids is to maintain a safer platelet count above 20000 to 50000. A reevaluation with a bone marrow biopsy/aspiration may be required to check for marrow failure. Patients with progressive marrow failure secondary to leukemic infiltration may benefit from CLL specific therapy. Also cytomegalovirus (CMV) reactivation has to be considered in the differential diagnosis in these patients as it is common in advanced stage of CLL and destroys platelets and megakaryocytes.^8^

Single agent rituximab or splenectomy may be tried in refractory cases. However, there is no randomized clinical trial which directly compares these two approaches. Rituximab is used in patients in whom splenectomy cannot be safely performed.^54^,^55^ Although rituximab is well tolerated, only 20% of patients achieved a sustained response.^55^ Low dose mycophenolate mofetil may be used after rituximab to prolong the response in selected patients.^56^

### Single agent monoclonal antibody therapy

Rituximab,^57^ Alemtuzumab^58^ and Veltuzumab^59^ have been reported to be effective in CLL associated AIT.^60^,^61^ Most of the published literature about rituximab is in primary immune thrombocytopenic purpura. Rituximab may be used in steroid refractory disease as a single agent, when treatment for CLL is not required. It may also be used in fludarabine induced autoimmunity.^61^ The dose of rituximab is 375mg per square meter given weekly for 4 weeks. A lower fixed dose of 100mg per week also has been studied, and has similar response rates.^62^,^63^

### Thrombopoietin agonist

Thrombopoietin (TPO), a ligand for c-mpl, stimulates committed megakaryocytic progenitors proliferation and induces maturation of megakaryocytes. Two thrombopoietin receptor agonists, romiplostim^64^ and eltrombopag^65^ were studied in multiple studies and are the only agents approved by FDA for the treatment of primary immune thrombocytopenic purpura. In a Danish retrospective registration study,^66^ Seven patients of secondary ITP (CLL is one of them, the others included Systemic lupus erythematosus, Evans syndrome, Human Immunodeficiency Virus infection and celiac disease ) were treated with TPO agonists. 57% responded with a platelet counts greater than 30,000 after 4 weeks.

TPO agonists are used for thrombocytopenia refractory to other therapies such as corticosteroids, intravenous immunoglobulin (IV Ig), single agent rituximab and splenectomy or when splenectomy is contraindicated.^67^,^68^ It has been suggested that these therapeutic modules should be used when a durable response is not obtained with other therapies and the platelet count continues to decline below 50,000.

The European Medicines Evaluation Agency approved TPOs for splenectomised patients who are refractory to other treatments. The American Society of Hematology recommendation for TPOs includes patients at risk of bleeding, patients in whom splenectomy is contraindicated, and patients who have failed at least one of therapies other than glucocorticoids.^43^ Thrombopoietins do not cause remission and continuous administration is required. Although TPOs are well-tolerated and no significant side effects are generally reported, studies have shown that they can increase bone marrow reticulin fibrosis and have increased risk of thrombocytosis.^69^ There are no trials comparing romiplostim and eltrombopag. However, higher incidence of portal vein thrombosis reported with eltrombopag in thrombocytopenia secondary to chronic liver disease. We suggest that the etiology of thrombocytopenia should be sought before using TPOs in patients with liver disease.^67^

### Splenectomy

Antibody and complement bound blood cells still have the ability to function normally. Spleen clears opsonized cells from circulation, and by reducing its rate, splenectomy may be an effective treatment. In a large series published by Vianelli *et al* in 2013 with a minimum of ten years follow up,^70^ there was a durable response to splenectomy. Out of 233 patients followed for 10 years, there was 88% response to splenectomy with 77 % complete response. The response was well-maintained, and free of any treatment in 59 % of patients. Splenic irradiation may be an alternative for patients in whom surgery is contraindicated.

Patients will require vaccination against encapsulated organisms including *Streptococcus pneumonia*, *Hemophilus influenza b*, and *Neisseria meningitides* at least 2–3 weeks before splenectomy.^71^–^73^ CLL patients are generally at increased risk of infections owing to defects in both cellular and humoral immune systems and qualitative and quantitative defects in B, T, and natural killer (NK) cells, neutrophils and the monocyte/macrophage system.^74^ Splenectomised patients have variable response to vaccination and immunization is most useful if used earlier in the disease process, and when immunoglobulin levels are better preserved.^74^ Considering that protein or conjugated vaccines are preferred for better immune response,^75^–^78^ it may be appropriate to routinely immunize all patients at the time of diagnosis, and before extensive immunosuppressive treatment has been given.

### Supportive care

In AIT patients with active bleeding, supportive care including platelet transfusions, antifibrinolytic agents such as tranexamic acid and eventually emergent surgical intervention may be required.

Patients on long term steroids are at risk of *Pneumocystis jiroveci* infection and prophylaxis is required. It is not clear that above which dose of steroids patients are considered to be at risk, but there is a general agreement that any dose above 20 milligrams per day has a higher risk for *Pneumocystis jiroveci*.^8^

### Autoimmune thrombocytopenia in the setting of advanced CLL

There is no standard treatment for this subgroup of patients. As described above, purine analogues and alkylating agents have a risk of inducing autoimmunity and should be avoided.

Two mostly studied chemo immunotherapy regimens in these patients include rituximab, cyclophosphamide and dexamethasone (RCD)^79^–^82^ and rituximab, cyclophosphamide, vincristine and prednisone (R-CVP).^83^ These two regimens have not been compared with each other in any randomized clinical trials.

In 2009, Kaufman *et al* reported RCD reported usage of RCD in 21 patients with progressive CLL and coexisting autoimmune cytopenias.^84^ Three Out of 21 patients had steroid resistant AIT and their nadir platelet counts were 1000, 1000 and 14000. The platelet counts increased to 408000, 161000 and 135000 after treatment with RCD in 9, 15, and 29 months respectively. The first patient who relapsed at 9 months was retreated with RCD and had 15 months’ response without any second relapse.

In a case control study of R-CVP by Bowen *et al*, 6 out of 20 studied patients had AIT.^83^ The median increase in platelet count was 106000 (range from 31000 to 216000), and median time to next treatment was 21.7 months. Patients with no response to R-CVP subsequently had a sustained complete response to splenectomy.

## Autoimmune Neutropenia

Autoimmune neutropenia in CLL requires a multidisciplinary management plan as these patients are at increased risk of atypical infections. Owing to both qualitative and quantitative defects in B, T, and NK cells both cellular and humoral arms of the adaptive immune systems are impaired in CLL. Monocyte/macrophage lineage is affected resulting in impairment of the innate immunity.^74^

When the absolute neutrophil count reaches below 500, risk of infection is increased, and it is important to consider treatment of AIN. AIN accompanied by fever of more than 100.4 F should be treated as neutropenic sepsis. Granulocyte colony stimulating factor (G-CSF) and prophylactic antibiotics are indicated in persistent neutropenia without fever. However, normalization of neutrophil count does not last long after discontinuation of G-CSF. Splenectomy was commonly used before the availability of G-CSF but the results are not encouraging.^85^

Sirolimus, cyclosporine and IVIg are reported to be helpful in AIN in several case reports.^85^–^87^ Rituximab and Alemtuzumab can provide long lasting remissions but the evidence is limited.^88^,^89^

Finally, it is very import to offer age appropriate vaccination to all CLL patients to prevent life threatening infections.

## Conclusion

Compared to cytopenias from the leukemic marrow involvement, autoimmune cytopenias have a better prognosis. The pathogeneses of AIT and AIN are not well understood, and lack of confirmatory tests is a diagnostic challenge. Since CLL associated cytopenias are not common, there is no randomized clinical trial on management of these conditions. Multicenter randomized clinical trials are necessary to enroll more patients in therapeutic trials.

## Figures and Tables

**Table 1:** Specific autoimmune disorders associated with Chronic Lymphocytic Leukemia.

Hematologic	Non-Hematologic
Autoimmune hemolytic anemia (AIHA)	Cold agglutinin disease (CAD)
Autoimmune thrombocytopenia (AIT)	Paraneoplastic pemphigus (PNP)
Autoimmune neutropenia (AIN)	Acquired angioedema (AAE)
Pure red cell aplasia (PRCA)	Anti-myelin associated glycoprotein polyneuropathy

**Table 2:** Facts and myths of Autoimmune disorders associated with Chronic Lymphocytic Leukemia.

Facts	Myths
Autoimmune hemolytic anemia (AIHA)	Hashimoto’s thyroiditis
Autoimmune thrombocytopenia (AIT)	Rheumatoid arthritis
Autoimmune neutropenia (AIN)	Systemic lupus erythematosus
Pure red cell aplasia	Polymyositis-dermatomyositis
Cold agglutinin disease (CAD)	Autoimmune glomerulonephritis
Paraneoplastic pemphigus (PNP)	Sjogren’s syndrome
Anti-myelin associated globulin polyneuropathy	vasculitis
Acquired angioedema (C1-INH deficiency)	Raynaud’s disease
	Autoimmune gastritis
	Ulcerative colitis
	Acquired hemophilia
	Acquired von Willebrand disease

**Table 3:** Differential Diagnosis of thrombocytopenia in Chronic Lymphocytic Leukemia.

**Chronic:** Drug Induced: Previous treatment with purine analogs, alkylating agents and monoclonal antibodies such as rituximab and alemtuzumab Autoimmunity Bone marrow infiltration by leukemic cells Splenomegaly Coexisting chronic infections such as hepatitis C, HIV and *Helicobacter pylori.*
**Acute:** Acute bacterial, viral and fungal infections; sepsis Recently used drugs: prescribed and over the counter medications.

**Table 4:** Diagnostic dilemmas and caveats to look for while diagnosing Autoimmune thrombocytopenia and Autoimmune Neutropenia.^38^,^46^

Autoimmune Thrombocytopenia	Autoimmune Neutropenia
Typical presentation: Sudden fall in platelet count to <100,000 *or* >50% drop from previous value *and* No evidence of splenomegaly, increased platelet destruction, recent chemotherapy *and* normal or increased megakaryocytes on bone marrow.	Typical presentation: Persistent unexplained neutropenia (Absolute neutrophil counts below 500) *and* no recent chemotherapy *and* bone marrow showing decreased or absent granulocyte precursors or maturation arrest. Positive anti neutrophil antibodies can sometimes be suggestive of autoimmunity.
*Watch for:*	*Watch for:*
If the patient is not anemic, thrombocytopenia is more likely to be AIT irrespective of the rate of fall. Spleen can be involved by CLL. Megakaryocytes can sometimes be difficult to appreciate in a bone marrow heavily infiltrated by CLL.	All other possible causes of neutropenia should be excluded carefully. Bone marrow sometimes may be difficult to appreciate if it is heavily infiltrated by CLL.

**Table 5:** Treatment regimens of Auto immune thrombocytopenia in patients who does **not** need treatment for their CLL.

Non Emergent Situations	
Corticosteroids :	
1) Oral prednisone	0.5 to 2 mg/kg per day
2) Oral dexamethasone	40 mg per day for 4 days (47)
Immunosuppressive agents	
1) Cyclosporine	2.5 3 mg/kg/day, after a starting dose of 5 mg/kg/d for six days, to maintain a therapeutic serum level between 200 and 400 ng/mL (48).
2) Mycophenolate Mofetil	1.5 2 g/day for at least 12 weeks (49)
3) Cyclophosphamide	Monthly 1.0 to 1.5 g/m^2^ Intravenous for 4 doses (51)
4) Azathioprine	150mg/day (53)
Monoclonal antibodies:	
1) Rituximab	375 mg/m^2^ per week or 100 mg fixed dose per week
2) Alemtuzumab	30 mg three times a week for 11 weeks (58)
Splenectomy	
Thrombopoetin receptor agonists:	
Romiplostim	2 microgram/kg/week for platelet count 10×10^9^/L or less and 2 microgram/kg every 2 weeks if platelet count is 11×10^9^/L to 50×10^9^/L. Once platelet count reach > 50×10^9^/L, the maintenance algorithm should be used: The dose should be increased by 1 μg/kg every week if 10×10^9^/L or less; increased by 1 μg/kg after 2 weeks if 11×10^9^/L to 50×10^9^/L; reduced by 1 μg/kg after 2 consecutive weeks at 201×10^9^/L to 400×10^9^/L; withheld if more than 400×10^9^/L and subsequent doses reduced by 1 μg/kg and start when count was less than 200×10^9^/L. The maximum allowed dose was 15 μg/kg (68)
Eltrombopag	50 mg/day if PLT<30,000 for 3 weeks and then If PLT still less than 50,000 increase the dose to 7 g mg/day(67)
**Emergent situations**	
1) Intravenous Immunoglobulin	1.0 gram/day (43)
2) Methyl Prednisone	1 gram/day for 3–4 doses(4)

**Table 6:** Treatment regimens of Autoimmune thrombocytopenia in patients who progressive CLL.

**RCD**	Rituximab 375mg/m^2^ IV on day 1; Cyclophosphamide 750mg/m^2^ on day 2; Dexamethasone 12 mg IV on days 1 and 2, and orally on days 3 through 7. These cycles were repeated every 3–4 weeks, depending upon recovery of blood counts.
**R-CVP**	Cyclophosphamide 750mg/m^2^, Vincristine 1.4 mg/m^2^ (maximum 2 mg) and Rituximab 375mg/m^2^ intravenously on day 1 and oral prednisone 40 mg/m^2^ days 1–5 of each 21 day cycle with the intent to treat with 6 cycles of therapy.
